# Clinical Outcomes of CADASIL-Associated *NOTCH3* Mutations in 451,424 European Ancestry Community Volunteers

**DOI:** 10.1007/s12975-018-0671-6

**Published:** 2018-10-18

**Authors:** Jane A. H. Masoli, Luke C. Pilling, George A. Kuchel, David Melzer

**Affiliations:** 1 Epidemiology and Public Health, Institute of Biomedical and Clinical Science, University of Exeter Medical School, Barrack Road, Exeter, EX2 5DW UK; 2 Healthcare for Older People, Royal Devon and Exeter NHS Foundation Trust, Barrack Road, Exeter, EX2 5DW UK; 3 UConn Center on Aging, University of Connecticut Health Center, 263 Farmington Avenue, Farmington, CT 06030-5215 USA

Cerebral autosomal dominant arteriopathy with subcortical infarcts and leukoencephalopathy (CADASIL) is a relatively rare hereditary small vessel disease resulting in neurological deficits [1]. Stroke and transient ischaemic attack (TIA) are reported in the majority with symptomatic CADASIL, driving presentation in 71% in a large case series, migraine is common (23%) and life expectancy is reduced [1, 2]. CADASIL can also contribute to vascular dementia. The exact prevalence of CADASIL is unknown as it is under-recognised even in specialist stroke settings, but the minimum prevalence is estimated at 2–5 in 100,000 [1].

Previous CADASIL studies have generally involved genotyping for patients presenting with repeated strokes, with several missense (amino-acid substituting) mutations in the *NOTCH3* gene reported [3]. Many, often rare, mutations have been linked to CADASIL. However, population-based estimates of the predictive value of *NOTCH3* missense mutations in community samples are unknown. With increasing availability of genotyping, including in clinical biobanks and direct to consumers, data are needed on the true penetrance of *NOTCH3* mutations.

**Fig. 1:**
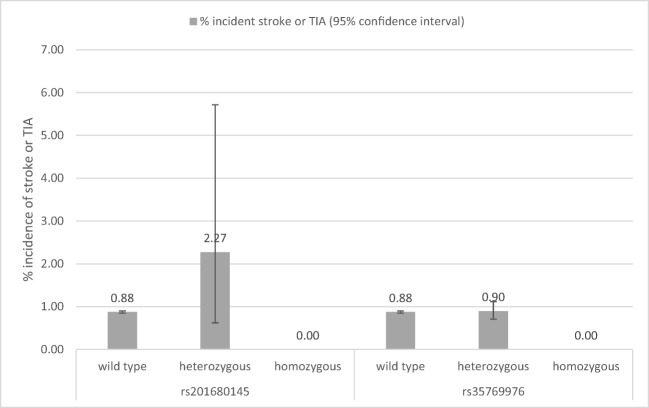
Graph of percentage with incident stroke or TIA during follow-up by *NOTCH3* variant genotype

We aimed to estimate cerebrovascular outcomes associated with all missense (amino-acid altering) likely pathogenic variants in *NOTCH3* in 451,424 participants of European descent in the UK Biobank (UKB) volunteer study. UKB community volunteers consented for genotyping and baseline (2006–2010) physiological measures, including systolic and diastolic blood pressure (BP), plus demographics and medical history [4]. The cohort was linked to national hospital inpatient and death certificate records and followed up for a mean of 7 years (maximum 10). We searched UKB Affymetrix Axiom array genotype data with imputation (v3, ~ 93million variants) for all amino-acid substituting variants in *NOTCH3* (*n* = 131), excluding low imputation quality (< 80%) and in linkage disequilibrium (R^2^ > 0.3), leaving 22 variants for follow-up. We used the Variant Effect Predictor (http://grch37.ensembl.org/Homo_sapiens/Tools/VEP, accessed: 17th May 2018) tools to prioritise variants with potential pathogenic effects; this process identified rs201680145 (p.Arg1231Cys: 0.04% mutation prevalence in UKB) and rs35769976 (p.Ala1020Pro; 0.96% mutation prevalence in UKB) only. Logistic regression tested cross-sectional associations between genotype and baseline BP, prevalent stroke or TIA, and prevalent Coronary Heart Disease (CHD). We used Cox proportional hazards regression models for all-cause mortality and Fine and Gray competing risk models for incident stroke, with all-cause mortality as the competing risk. Those with prior strokes were included in the analysis of incident strokes as CADASIL is linked to recurrent strokes. Models were adjusted for age, sex and technical genetic covariates (including genetic principal components to account for population admixture). Analyses were conducted using Stata v14.1. The rs201680145 variant has been reported as pathogenic for CADASIL by two sources [5]. There were 176 UKB participants heterozygous for the missense A allele of rs201680145 (p.Arg1231Cys/p.R1231C, prevalence 0.04% of 451,424) and no homozygous participants. Mean age was 56.8 years (SD 7.7, median 58, IQR 13). rs201680145 heterozygotes had modest increases in baseline systolic (3.12 mmHg; 95% confidence interval 0.28 to 5.96) and diastolic blood pressure (BP) (2.02 mmHg, 0.39 to 3.64), compared to no mutations. Figure 1 shows the percentage of subjects developing incident stroke or TIA, by *NOTCH3* variant genotype. During the follow-up period, four of 176 A allele rs201680145 heterozygotes experienced incident strokes or TIAs (2.27%), compared to 0.88% (3874/442,613) with no mutation: sub-HR 3.21, 1.20 to 8.56; *p* = 0.020. Incident CHD or all-cause mortality was not associated with rs201680145.

There were 8596 heterozygotes for rs35769976 and only 44 G allele homozygotes (0.01% of 451,424), so we grouped homozygotes and heterozygotes. The mean age of the sample was 56.8 years (SD 8.0, median 58, IQR 12). Participants with at least one rs35769976 G allele had a slightly increased diastolic BP (coefficient 0.24, 0.0079 to 0.47; *p* value = 0.043). There were no strokes or TIAs in the G allele homozygotes and 77 in heterozygotes. The rs35769976 variant was not associated with incident stroke or TIA, CHD or all-cause mortality.

In a European Ancestry community volunteer biobank, we have shown that only two potentially pathogenic *NOTCH3* missense variants were ascertained in array genotyping data with imputation. rs201680145, previously reported as pathogenic by two sources [5], was associated with elevated systolic and diastolic BP, plus substantially increased risk of incident stroke or TIA. However, the great majority of rs35769976 carriers did not have a stroke or TIA, suggesting that this mutation is associated with a milder clinical course. If these mutations were associated with high rates of serious strokes, we would expect prevalence of the mutations to be lower at older ages, but neither mutation was associated with age in UK biobank. A limitation of our analysis is that the maximum age at end follow-up was 80 years. However, as the cohort included those between 41 and 70 years at baseline, with up to 10-year follow-up, the important time for early strokes is captured. In addition, while 10-year follow-up is longer than in most reported CADASIL studies, data on even longer follow-ups would be helpful. This would also allow analysis of repeat events in individuals, as numbers with recurrent stroke or TIA in this cohort were too small to analyse.

This study suggests that potentially pathogenic *NOTCH3* missense mutations seen in large European ancestry array genotyped biobanks have relatively limited CADASIL risk profiles. Caution is needed in responding to incidental data on these mutations in community volunteer samples. More work is needed to establish whether co-factors (including higher blood pressures) modify risks of clinical outcomes.
